# LUMIADE 3: Therapeutic Equivalence of Denosumab Biosimilar FKS518 to Reference Product in Postmenopausal Osteoporosis

**DOI:** 10.1210/jendso/bvaf164

**Published:** 2025-10-22

**Authors:** Ewa Krecipro-Nizinska, Wojciech Pluskiewicz, Jerzy Supronik, Peter Szeles, Corinne Petit-Frere, Elsa Varone, Serge Ferrari, Ivo Valter

**Affiliations:** Centrum Medyczne FutureMeds Wroclaw, Wrocław 53-673, Poland; Department and Clinic of Internal Diseases, Diabetology and Nephrology, Metabolic Bone Diseases Unit, Faculty of Medical Sciences in Zabrze, Medical University of Silesia, Katowice 41-800, Poland; ZDROWIE Osteo-Medic s.c., L. i-A. Racewicz, A. i J. Supronik, Białystok 15-351, Poland; Department of Clinical Development, Fresenius Kabi SwissBioSim, CH-1262 Eysins, Switzerland; Department of Clinical Development, Fresenius Kabi SwissBioSim, CH-1262 Eysins, Switzerland; Department of Clinical Development, Fresenius Kabi SwissBioSim, CH-1262 Eysins, Switzerland; Division of Bone Diseases, Geneva University Hospital and Faculty of Medicine, University of Geneva, CH-1211 Geneva, Switzerland; Nordestmedical Center, Center for Clinical and Basic Research AS, Tallinn 10128, Estonia

**Keywords:** denosumab, FKS518, postmenopausal, osteoporosis, biosimilarity, efficacy

## Abstract

**Objectives:**

To demonstrate therapeutic equivalence of the proposed denosumab biosimilar FKS518 to the originator denosumab (US-licensed Prolia®, reference product), a potent antiresorptive biologic that increases bone mineral density (BMD) and reduces the risk of fractures, in women with postmenopausal osteoporosis.

**Methods:**

This 78-week double-blind, controlled, randomized, multicenter, multiple-dose, 2-arm, parallel-group study compared the efficacy (BMD), pharmacodynamic (bone biomarkers), safety, tolerability, and immunogenicity profiles of FKS518 with those of reference denosumab in women with postmenopausal osteoporosis. Primary—percentage change from baseline to 52 weeks in lumbar spine BMD and area under the effect curve from baseline to week 26 of serum C-terminal cross-linking telopeptide of type 1 collagen—and secondary results from the 52-week core treatment period are reported here.

**Results:**

Postmenopausal women with osteoporosis were randomized to receive 60 mg of FKS518 (n = 277) or reference denosumab (n = 276) every 26 weeks. Demographics, baseline characteristics and medical history were similar between treatment groups. Therapeutic equivalence of FKS518 and reference denosumab was demonstrated for efficacy and pharmacodynamic characteristics. All sensitivity analyses, supportive estimands, secondary efficacy, and pharmacodynamic endpoint analyses consistently showed similarity between the 2 products. Safety outcomes were consistent with the known safety profile of denosumab and were comparable between FKS518 and reference denosumab. Immunogenicity was infrequently observed and similar between the FKS518 and the reference denosumab groups.

**Conclusion:**

This study demonstrated therapeutic equivalence of, and comparable pharmacokinetics, safety, and immunogenicity profiles between FKS518 and reference denosumab, completing the clinical evidence to propose FKS518 as a biosimilar to denosumab.

Key Messages What is already known on this topic:

- Denosumab is a potent antiresorptive agent that reduces the risk of fractures and improves bone mineral density (BMD) in patients with osteoporosis, but its use may be limited by the high price of the originator denosumab product compared with other commonly used osteoporosis therapies.

What this study adds:

- Therapeutic equivalence and comparable pharmacodynamics, safety, and immunogenicity profiles were demonstrated between the proposed denosumab biosimilar FKS518 and the originator denosumab (US-licensed Prolia^®^).

How this study might affect research, practice, or policy:

- Improved access to a biosimilar denosumab such as FKS518 may benefit patients at increased risk of fractures and healthcare systems by potentially offering cost savings.

Osteoporosis is a chronic condition characterized by low bone density and deterioration of skeletal integrity that leads to increased bone fragility and risk of fractures [1]. It may develop because of several pathophysiological changes including postmenopausal estrogen loss and age-related deterioration of skeletal structure, underlying diseases, and some treatments [1, 2].

A pivotal regulator of bone metabolism is the receptor activator of nuclear factor kappa B (RANK) ligand (RANKL) signaling pathway [3], which modulates osteoclastic formation, function, and survival [3-5]. Production of RANKL is increased when estrogen levels are decreased, which leads to increased bone resorption [6]. Blocking of RANKL–RANK signaling on osteoclasts therefore reduces bone resorption, increases bone mineral density (BMD) in patients with osteoporosis, and protects against bone loss and skeletal-related events associated with bone metastases in people with solid tumors or multiple myeloma [6].

Denosumab is the first approved fully human monoclonal immunoglobulin (Ig)G2 antibody that inhibits RANKL [7, 8], reduces the risk of fractures and improves BMD. It is an effective anti-osteoporosis therapy for patients at high risk of fractures, and its effectiveness delays or prevents pathological fractures and other skeletal-related events in patients with advanced malignancies that involve the bone [7-11].

Denosumab is available in the European Union (EU) and the United States (US) as Prolia^®^ and Xgeva^®^, respectively [4, 5, 12, 13]. These products contain the same active substance (denosumab) and qualitatively the same excipients, and have the same mechanism of action, but differ in strength, presentation, and indications for use. Among other indications, Prolia^®^ is approved for the treatment of osteoporosis in postmenopausal women at increased risk of fractures. However, although osteoporotic fractures are a major cause of disability and healthcare costs [14-16], the use of denosumab may be limited by the high price of the originator denosumab product compared with other commonly used therapies for osteoporosis [10, 11]. A denosumab biosimilar therefore may be valuable, with the potential to lead to cost savings and improved access to treatment [17-21].

Biosimilar medicines are approved based on a rigorous stepwise exercise to establish similarity between a biosimilar and the reference biologic (originator) [22-25]. FKS518 is being developed as a proposed denosumab biosimilar to Prolia^®^/Xgeva^®^. The pharmacokinetic (PK) similarity of FKS518 60 mg to US-licensed denosumab 60 mg (Prolia^®^) has been demonstrated in a phase 1 comparative PK, pharmacodynamic (PD), safety, and immunogenicity study in healthy male subjects [26]. In this paper, we describe the key results of LUMIADE 3, a phase 3 study that compared the efficacy, PD, immunogenicity, and safety of FKS518 60 mg and US-licensed denosumab 60 mg (Prolia^®^; reference denosumab) in postmenopausal women with osteoporosis. The results of this study contribute to the totality of evidence needed to demonstrate similarity between FKS518 and reference denosumab.

## Methods

This was a double-blind, controlled, randomized, multicenter, multiple-dose, 2-arm, parallel-group study to compare the efficacy, PD (bone biomarkers), safety, tolerability, and immunogenicity of the proposed denosumab biosimilar FKS518 with those of reference denosumab (US-licensed Prolia^®^) in ambulatory women with postmenopausal osteoporosis. The study included a screening period of maximum 28 days, a 52-week core treatment period, and a 26-week second (transition) treatment period. This report describes results from the core treatment period, which includes evaluation of the primary study objective. Results from the transition treatment period that evaluated the potential impact of switching from reference denosumab to FKS518 at week 52 on efficacy, safety, and immunogenicity will be reported separately.

**Figure 1.**
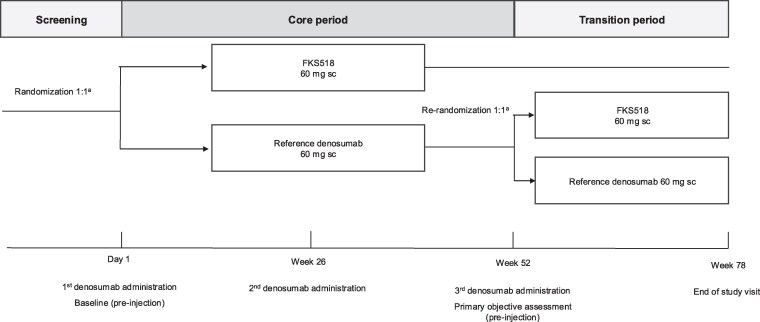
Study design. ^a^Randomization was stratified by age (<65 years, ≥65 years) and prior bisphosphonate therapy (yes, no). Abbreviation: sc, subcutaneous.

The study enrolled patients from 64 investigative sites in Europe (Bulgaria, Czechia, Estonia, Georgia, Hungary, and Poland) and was conducted between June 2021 and August 2023. Using an interactive response technology system, eligible participants were randomized 1:1 to receive either FKS518 or reference denosumab, administered at a dose of 60 mg subcutaneously into the abdomen using a single-use pre-filled syringe, starting on day 1 and then every 26 weeks (6 months) for a total of 3 administrations (Fig. 1). Randomization was stratified by age (<65 years; ≥65 years) and prior bisphosphonate therapy (Yes/No). Participants were instructed to take calcium 1000 mg and vitamin D supplementation ≥400 IU daily, corresponding to adequate daily supplementation [4].

The study was conducted in compliance with the Declaration of Helsinki and all relevant regulations (International Council for Harmonisation E6 [R2], European Union [EU] Directive 2005/28/EC, and EU Regulation 536/2014). In addition, this study adhered to all local regulatory requirements and requirements for data protection. Independent Ethics Committee (IEC) review and approval was obtained in each participating country before any patients were enrolled. Since the study was conducted across multiple countries, each site had its own local IEC responsible for ethical oversight. For example, the principal investigator of LUMIADE 3 was affiliated with the Center for Clinical and Basic Research AS in Tallinn, Estonia, and the IEC was the Estonian Committee on Bioethics and Human Research (Eesti bioeetika ja inimuuringute nõukogu, which is affiliated with the Ministry of Social Affairs of Estonia). The study is registered (EudraCT Number: 2020-004422-31 and NCT04934072).

### Objectives

The study objective was to demonstrate therapeutic equivalence of the proposed denosumab biosimilar FKS518 to reference denosumab in women with postmenopausal osteoporosis. The study included 2 powered endpoints to fulfill the requirements of both the US (Food and Drug Administration) and European regulatory agencies (European Medicines Agency): percentage change from baseline (%CfB) in lumbar spine bone mineral density (LS-BMD) and area under the effect curve from baseline to week 26 (AUEC_(0-W26)_) of serum C-terminal cross-linking telopeptide of type 1 collagen (CTX). There were 3 main estimands. Estimand 1 assessed the treatment effect on the %CfB in LS-BMD at week 52 for all participants randomly assigned to treatment using an intention-to-treat (ITT) approach (treatment policy estimand). Estimand 2 used the same endpoint but assessed the treatment effect, as if all participants had taken treatment as prescribed with no change to concomitant or background medication affecting bone and with no bone-affecting adverse events (AEs) (hypothetical continuing per protocol [PP] estimand). Estimand 3 assessed the treatment effect on the PD endpoint CTX AUEC_(0-W26)_, using the same hypothetical continuing PP approach as Estimand 2. Secondary objectives compared PD, safety, tolerability, and immunogenicity of FKS518 to the reference denosumab.

### Participants

Eligible participants were women with osteoporosis aged ≥55 to ≤85 years, with confirmed postmenopausal status, body mass index ≥18 to ≤32 kg/m^2^, a LS-BMD T-score ≤ −2.5 and ≥ −4.0 as measured by central assessment of dual energy x-ray absorptiometry (DXA), and who had at least 2 vertebrae in the L1–L4 region and at least 1 hip joint evaluable by DXA. Women were excluded if they had concomitant medical disorders or medications that could pose a risk to their safety or affect study assessments or procedures (Supplementary file [27]).

All participants provided written informed consent before entering the study.

### Study Procedures

Efficacy assessments were performed at baseline and at week 52. BMD was measured at the lumbar spine (LS-BMD, including L1 through L4 vertebrae, duplicate scans) and the left proximal femur (femoral neck and total hip BMD) using DXA (Lunar or Hologic) scans. All DXA scan data were analyzed by a central imaging vendor. PD measurements (CTX and procollagen type 1 N-terminal propeptide [P1NP]), safety, and immunogenicity assessments were performed regularly throughout the 52 weeks (Table S1 [27]). The CTX assay used a modified Crosslaps^®^ ELISA kit (Immunodiagnostic Systems Cat# AC-02F1, RRID:AB_2923399) and P1NP measurements were performed using an electrochemiluminescent sandwich immunoassay in an automated Roche COBAS 8000 E602 analyzer (Roche Cat# 03141071190, RRID:AB_2782967). Concentrations of bone biomarkers were measured using blood samples collected in the morning after an overnight fast.

Safety assessments were based on the recording, reporting, and analysis of treatment-emergent adverse events (TEAEs, including serious TEAEs [SAEs]), physical examination findings, vital signs, 12-lead electrocardiogram tracing, laboratory tests (clinical chemistry, hematology, and urinalysis), local tolerability, and signs and symptoms suggestive of hypersensitivity, as judged by the Investigator. Adverse events were coded using the standardized Medical Dictionary for Regulatory Activities (MedDRA) version 24.0. Drug-related hypersensitivity/allergic reactions (Common Terminology Criteria for Adverse Events [CTCAE] Grade ≥ 3 or reported as SAEs), and TEAEs leading to study drug discontinuation or study withdrawal were pre-defined as TEAEs of special interest (AESI). All confirmed COVID-19 infections were considered as “otherwise medically important” events and were classified as SAEs, regardless of severity or meeting other seriousness criteria, given the limited knowledge of the infection at the time of the study. Fractures were not evaluated systematically by mandatory scans at given time points for each patient, instead those identified were recorded as adverse events.

Immunogenicity testing used a multi-tiered approach. Serum immunogenicity samples were used to determine the incidence of antidrug antibodies (ADA), ADA titers, and incidence of neutralizing antibodies (NAb) using validated methods. A sequential bridging electrochemiluminescent immunoassay, utilizing denosumab detection and capture antibodies (denosumab antibody AbD26781_hIgG1: Bio-Rad Cat# HCA283, RRID:AB_3712665 and anti-denosumab antibody AbD26295_hIgG1: Bio-Rad Cat# HCA280, RRID:AB_3712666), was used to detect ADA and a competitive ligand binding assay evaluated NAb (denosumab biosimilar targeting RANKL: FKS518: RRID:AB_3714756).

### Statistical Analyses

A sample size of 526 (263 patients per group) randomized patients was determined to provide at least 90% power to demonstrate equivalence between the 2 treatments for the %CfB in LS-BMD at week 52, with equivalence intervals of (−1.45%, +1.45%) and a type I error of 2.5%, assuming a maximum 0.2% difference between products, a common standard deviation (SD) of 4.0% and a 15.0% dropout rate. This sample size guaranteed at least 99% power to show equivalence between treatments for the AUEC_(0-W26)_ of serum CTX with equivalence intervals of (0.89, 1.12) assuming no difference between groups. All randomized patients were included in the ITT analysis set. The safety analysis set included all treated patients. The PP and PD analysis sets included all treated subjects with no important protocol deviations impacting efficacy or the PD endpoint, respectively. For more details see Table S2 [27]. All analyses used SAS Version 9.4 or higher.

For Estimand 1, the main analysis consisted of 2 separate 1-sided tests (noninferiority/nonsuperiority) at alpha = 0.05 level. All available data were included in the analysis (ITT principle) and missing LS-BMD data at week 52 were imputed “under the null” method where participants treated with FKS518 were assumed to worsen from missing at random by the amount of the noninferiority (−1.45)/nonsuperiority (+1.45) margin. The main estimate was the difference in least squares mean (LSM) %CfB at week 52 in LS-BMD between FKS518 and reference denosumab, with its 90% CI, calculated using an analysis of covariance (ANCOVA) model with %CfB at week 52 in LS-BMD as response variable, treatment, and randomization stratification factors as fixed effects, and baseline LS-BMD as a covariate. The ANCOVA model was run separately for the noninferiority and nonsuperiority tests, based on the different imputation rules to be used.

Noninferiority and nonsuperiority of FKS518 to reference denosumab were demonstrated if the lower bound of the 90% CI for the difference in LSM %CfB to week 52 in LS-BMD was above −1.45% and the upper bound was below +1.45%, respectively. FKS518 was considered equivalent to reference denosumab if both noninferiority and nonsuperiority criteria were met.

For Estimand 2, LS-BMD assessments were censored when affected by intercurrent events and were imputed by means of multiple imputation applying a hypothetical PP strategy. Missing and censored measurements were imputed using data from patients with similar baseline characteristics who had no intercurrent events and assuming missing at random. The main estimate was the LSM difference in LS-BMD %CfB between FKS518 and reference denosumab, with its 95% CI calculated using same ANCOVA model as described above for Estimand 1. Equivalence was shown if the 95% CI for the difference between treatments lay entirely within the equivalence interval of (−1.45%, +1.45%).

For Estimand 3, CTX data points were censored for the duration of intercurrent events. A hypothetical strategy was followed where all missing AUEC_(0-W26)_ CTX values were imputed using a per protocol scenario (as if the subject had continued to follow the protocol and did not have an intercurrent event) assuming MAR. The main estimate was the ratio of the geometric LSM of AUEC_(0-W26)_ for FKS518 to reference denosumab with its 95% CI, calculated using an ANCOVA with the natural log of AUEC_(0-W26)_ CTX as response variable, treatment group and randomization stratification factors as fixed effects, and the natural log of baseline serum CTX concentration as a covariate. The results were then back-transformed to the original scale. FKS518 was considered equivalent to reference denosumab in terms of PD if the 95% CI for the ratio of LSM of AUEC_(0-W26)_ CTX lay entirely within the equivalence interval of (0.89, 1.12).

No adjustment for multiplicity was needed as all relevant estimands had to meet the success criterion to conclude equivalence for a given regulatory agency.

Sensitivity analyses were performed for the 3 main estimands, including tipping point analysis.

Additional supportive estimands were defined using a complete case approach for both %CfB in LS-BMD at week 52 (on the PP analysis set) and AUEC_(0-W26)_ CTX (on the PD analysis set).

Secondary efficacy and PD endpoints were summarized descriptively on the ITT analysis set.

### Safety and Immunogenicity

Safety data were listed and summarized using appropriate descriptive statistics.

ADA and NAb incidence, as well as ADA titer over time (not presented here), were summarized descriptively by treatment group and by scheduled visit and overall; the denominator for each visit was the number of patients with a valid ADA result at that visit.

## Results

**Figure 2.**
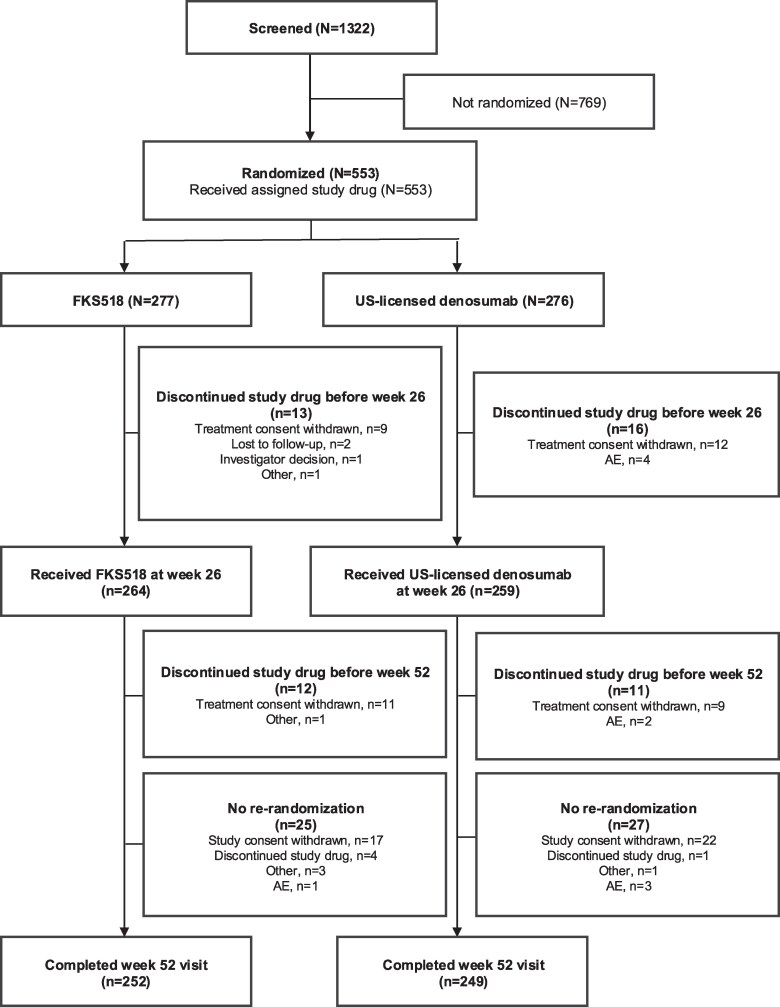
Study subject disposition. A patient could have had more than 1 reason for discontinuation. Abbreviations: AE, adverse event; US, United States.

Of the 1322 screened women with postmenopausal osteoporosis, a total of 553 were randomized and received either FKS518 (*n* = 277) or reference denosumab (*n* = 276) (Fig. 2). Similar proportions of participants in the FKS518 and the reference denosumab groups received their second injection at week 26 (*n* = 264 [95.3%] and *n* = 259 [93.8%], respectively). The main reason for discontinuing either study treatment during the study was withdrawal of consent for treatment.

Overall, 254 (45.9%) of the included women were aged <65 years, and their baseline weight was between 43 and 96 kg. All participants were White. Demographics, baseline characteristics, and medical history were similar between the FKS518 and the reference denosumab groups (Table 1). Baseline LS-BMD, femoral neck BMD, and total hip BMD were also balanced between the 2 groups (Table 1). Prior medications for postmenopausal osteoporosis had been taken by 36.1% and 34.1% of the FKS518 and the reference denosumab groups, respectively; most commonly monotherapy with vitamin D and analogues (17.4% overall), bisphosphonates (alone or in a combination; 11.9% overall), calcium monotherapy (11.0%), and calcium combinations with vitamin D and/or other drugs, excluding bisphosphonates (9.2%). The proportions of participants in each treatment group were similar with respect to the number and types of concomitant disorders, most commonly hypertension (38.9% overall), osteoarthritis (19.0%), and hypercholesterolemia (18.1%).

**Table 1.** Patient demographics and baseline characteristics

Variable	FKS518 (*N* = 277)	Reference denosumab (*N* = 276)
Female, *n* (%)	277 (100)	276 (100)
Age, years	65.2 (6.4)	65.8 (6.5)
Age group, *n* (%)		
<65 years	128 (46.2)	126 (45.7)
≥65 years	149 (53.8)	150 (54.3)
Prior bisphosphonate therapy, *n* (%)	32 (11.6)	34 (12.3)
Weight, kg	63.5 (9.8)	62.7 (8.8)
BMI, kg/m^2^	24.9 (3.5)	24.6 (3.3)
History of fracture, *n* (%)	74 (26.7)	78 (28.3)
Family history of hip fracture*^a^*, *n* (%)	29 (10.5)	26 (9.4)
Low dietary calcium intake*^b^*, *n* (%)	162 (58.5)	164 (59.4)
Sedentary lifestyle*^b^*, *n* (%)	65 (23.5)	77 (27.9)
Time since diagnosis, years	2.5 (3.9)	2.9 (4.8)
Age at menarche, years	13.8 (1.4)	13.7 (1.5)
Age at menopause, years	49.4 (4.8)	48.6 (5.1)
Number of pregnancies	2.3 (1.5)	2.2 (1.5)
LS-BMD by DXA,*^a^* g/cm^2^	0.787 (0.064)	0.793 (0.060)
LS-BMD T-score by DXA*^a^*	−3.015 (0.406)	−3.012 (0.395)
BMD at femoral neck by DXA, g/cm^2^	0.705 (0.095)	0.722 (0.094)
BMD at total hip by DXA, g/cm^2^	0.773 (0.097)	0.784 (0.090)
Serum CTX, pg/mL	558.7 (248.3)	533.2 (276.2)
Serum P1NP, ng/mL	65.0 (27.0)	62.3 (24.4)

Data presented are mean (SD) unless otherwise stated. Analysis set: intention-to-treat.

LS-BMD was determined as the average of adjusted and corrected lumbar duplicate DXA scans; for femoral neck and hip BMD, corrected values were determined.

Abbreviations: BMD, bone mineral density (corrected); BMI, body mass index; CTX, C-terminal cross-linking telopeptide of type 1 collagen; DXA, dual energy x-ray absorptiometry; LS-BMD, lumbar spine bone mineral density; P1NP, procollagen type 1 N-terminal propeptide.

^*a*^Family history of hip fracture refers to parental history.

^*b*^Based on investigator judgment.

### Primary Endpoints

Clinically relevant increases from baseline in LS-BMD were evident at week 52 in both treatment groups.

**Figure 3.**
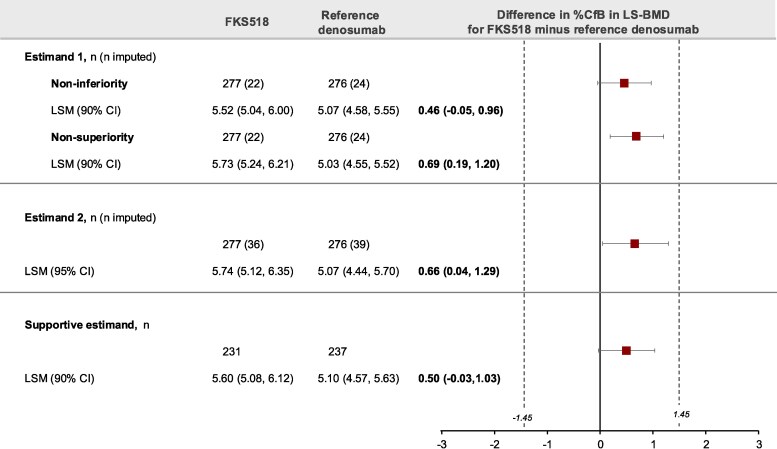
Percentage change from baseline in LS-BMD by DXA at week 52. Data presented are the difference in LSM %CfB of LS-BMD by DXA at week 52. LS-BMD was determined as the average of adjusted and corrected lumbar duplicate DXA scans. Equivalence margins shown by dotted lines. Estimand 1, noninferiority: all missing assessments are imputed and then a shift of −1.45 applied; nonsuperiority: a shift of 1.45 is applied. Shifts are added only to the FKS518 treatment group assessments and assume that participants treated with FKS518 worsened from missing at random by the amount of the noninferiority (−1.45) and nonsuperiority (+1.45) margin. Estimand 2: missing baseline and missing LS-BMD at week 52 are imputed by a multiple imputation procedure, assuming missing data at random. Supportive estimand: complete cases in the per protocol analysis set. Abbreviations: %CfB, percentage change from baseline; DXA, dual energy x-ray absorptiometry; LS-BMD, lumbar spine bone mineral density; LSM, least squares mean; n, number of subjects.

Equivalence in efficacy between FKS518 and reference denosumab was demonstrated for both Estimands 1 and 2 (Fig. 3). For Estimand 1, the lower bound of the 90% CI for the noninferiority test (−0.05) was above −1.45, and the upper bound of the 90% CI for the nonsuperiority test (1.20) was below 1.45. For Estimand 2, the 95% CI for the difference in %CfB in LS-BMD at week 52 (0.04%, 1.29%) was fully included within the equivalence interval (−1.45%; +1.45%).

**Figure 4.**
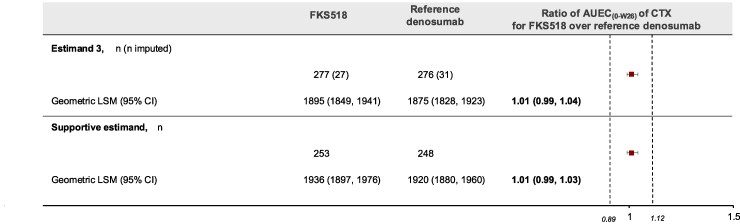
Analysis of the ratio of geometric least squares means of AUEC(0-W26) of CTX (mg*h/L). Data presented are the ratio of geometric LSM of AUEC(0-W26) CTX. Equivalence margins shown by dotted lines. Estimand 3: missing outcome values were imputed by means of a multiple imputation approach assuming missing at random. Supportive estimand: complete cases in the pharmacodynamic analysis set. Abbreviations: AUEC, area under the effect curve; CTX, C-terminal cross-linking telopeptide of type 1 collagen; LSM, least squares mean; n, number of subjects.

Equivalence in PD was demonstrated for Estimand 3, with the 95% CIs of the FKS518/reference denosumab geometric LSM ratio for AUEC_(0-W26)_ CTX (0.99,1.04) fully included within the equivalence interval of (0.89, 1.12) (Fig. 4).

Results from the sensitivity analyses and supportive estimands further supported the conclusions of the primary analyses.

### Secondary Endpoints

Similar %CfB in femoral neck and total hip BMD and similar %CfB reductions in serum CTX and P1NP were observed for the FKS518 and the reference denosumab groups at week 52 (Table 2).

**Table 2.** Percentage change from baseline at week 52 in bone mineral density and bone turnover biomarkers

Percentage change from baseline at week 52	FKS518 (*N* = 277) Mean (SD)	The reference denosumab (*N* = 276) Mean (SD)
LS-BMD	(*n* = 255)	(*n* = 252)
	5.67 (3.27)	5.04 (3.62)
Femoral neck BMD	(*n* = 257)	(*n* = 253)
	2.43 (3.72)	2.20 (2.97)
Total hip BMD	(*n* = 257)	(*n* = 253)
	3.11 (2.53)	3.00 (2.62)
Serum CTX	(*n* = 249)	(*n* = 246)
	−69.90 (53.74)	−64.29 (47.03)
Serum P1NP	(*n* = 249)	(*n* = 246)
	−65.48 (23.75)	−62.10 (37.01)

Analysis set: intention-to-treat.

Abbreviations: BMD, bone mineral density assessed by DXA (data collected in g/cm^2^); CTX, C-terminal cross-linking telopeptide of type 1 collagen (data collected in pg/mL); DXA, dual energy x-ray absorptiometry; LS-BMD, lumbar spine bone mineral density assessed by DXA; N, number of participants in the intention-to-treat analysis set; n, number of patients with no missing values; P1NP, type 1 N-terminal propeptide (data collected in ng/mL).

### Safety

Over the 52-week core study period, 1196 TEAEs were reported in a total of 374 (67.6%) participants: 185 (66.8%) participants in the FKS518 group and 189 (68.5%) in the reference denosumab group (Table 3). The most common TEAEs were COVID-19 followed by nasopharyngitis, upper respiratory tract infection, urinary tract infection, and headache (Table 3). Treatment-related TEAEs were reported in similar proportions of the FKS518 group (9.0%) and the reference denosumab group (11.2%). The most common treatment-related TEAE was arthralgia (1.4% of each treatment group).

**Table 3.** Treatment-emergent adverse events

Adverse event	FKS518 (*N* = 277) *n* (%) m	Reference denosumab (*N* = 276) *n* (%) m	Total (*N* = 553) *n* (%) m
TEAE	185 (66.8) 583	189 (68.5) 613	374 (67.6) 1196
Treatment-related TEAE	25 (9.0) 50	31 (11.2) 44	56 (10.1) 94
SAE	43 (15.5) 46	50 (18.1) 54	93 (16.8) 100
Treatment-related SAE	1 (0.4) 1	0	1 (0.2) 1
TEAE Grade ≥3	7 (2.5) 8	11 (4.0) 15	18 (3.3) 23
Treatment-related TEAE Grade ≥3	0	0	0
TEAE Grade ≥4	0	1 (0.4) 1	1 (0.4) 1
Treatment-related TEAE Grade ≥4	0	0	0
AESI*^a^*	0	7 (2.5) 11	7 (1.3) 11
Treatment-related AESI*^a^*	0	0	0
TEAE leading to treatment withdrawal	0	6 (2.2) 10	4 (0.7) 4
Treatment-related TEAE leading to treatment withdrawal	0	0	0
TEAE leading to discontinuation from the study	0	7 (2.5) 11	7 (1.3) 11
Treatment-related TEAE leading to discontinuation from the study	0	0	0
TEAE leading to death	0	0	0
ISR	1 (0.4) 2	2 (0.7) 3	3 (0.5) 5
Serious ISR	0	0	0
TEAE involving fracture	3 (1.1) 3	9 (3.3) 10	12 (2.2) 13
TEAE fracture involving the spine	1 (0.4) 1	0	1 (0.2) 1
TEAE fracture involving the femur and hip	0	1 (0.4) 2	1 (0.2) 2
Most common TEAEs*^b^*			
Infections and infestations	118 (42.6)	131 (47.5)	249 (45.0)
COVID-19	32 (11.6)	41 (14.9)	73 (13.2)
Nasopharyngitis	26 (9.4)	33 (12.0)	59 (10.7)
Upper respiratory tract infection	23 (8.3)	30 (10.9)	53 (9.6)
Urinary tract infection	18 (6.5)	23 (8.3)	41 (7.4)
Nervous system disorders	24 (8.7)	34 (12.3)	58 (10.5)
Headache	13 (4.7)	14 (5.1)	27 (4.9)

Analysis set: safety.

Treatment-emergence was defined as adverse events that began or increased in severity or frequency on or after the date of first study drug administration up to the early termination/end of study visit. Treatment-relatedness was assessed by the investigator.

Abbreviations: AESI, TEAE of special interest; ISR, injection site reaction; m, number of events; n, number of patients; NCI-CTCAE, National Cancer Institute—Common Terminology Criteria for Adverse Events; SAE, serious TEAE; TEAE, treatment-emergent adverse event.

^*a*^AESI were drug-related hypersensitivity/allergic reactions (NCI-CTCAE Grade ≥3 or reported as SAEs) and TEAEs leading to treatment discontinuation or withdrawal from the study during the core treatment period.

^*b*^TEAEs occurring in ≥5% of patients in either treatment group.

SAEs were also reported in a similar proportion of each treatment group (15.5% in the FKS518 group and 18.1% in the reference denosumab group), with COVID-19 infections being the most prevalent (80.0% of all SAEs), also occurring in similar proportions of both treatment groups. None of the non-COVID-19 SAEs were reported for more than one patient, except for balance disorder (2 patients; 1 in each treatment group). Only 1 SAE was considered treatment-related (a COVID-19 infection in an FKS518 recipient). No TEAEs leading to death were reported.

COVID-19 infections did not affect the type or incidence of other TEAEs reported by either denosumab group. Most TEAEs were Grade 1 or 2; only a low proportion of participants experienced Grade ≥3 TEAEs (2.5% in FKS518 group and 4.0% in the reference denosumab group), none of which were considered treatment-related. Only 1 participant (in the reference denosumab group) experienced a Grade ≥4 TEAE (ovarian cancer). Few patients experienced TEAEs leading to treatment interruption in the FKS518 and the reference denosumab groups (3 [1.1%] and 1 [0.4%], respectively); with only 1 event (dental fistula in a FKS518 recipient) considered treatment-related. Osteonecrosis of the jaw was not reported during the study.

AESIs were reported in no participants in the FKS518 group and 7 participants in the reference denosumab group (2.5%; all qualified as AESI as they were TEAEs leading to discontinuation from the study), with none considered treatment-related.

Low proportions of participants reported at least one injection site reaction after the first and second injections, with no notable differences between the FKS518 (0.4%) and the reference denosumab (0.7%) groups.

TEAEs involving any fracture occurred infrequently, being reported in 3 participants (1.1%) in the FKS518 group (involving the spine in 1 patient) and 9 (3.3%) in the reference denosumab group (involving the femur in 1 patient).

### Immunogenicity

The overall ADA incidence during the 52-week period was low (1.1% with FKS518 and 2.2% with reference denosumab) and the overall incidence of NAbs against denosumab was very low and similar in each treatment group (1 patient [0.4%] in each group) (Table 4). Median ADA titers were also low (no titers were above the assay minimum required dilution), with no meaningful differences in efficacy or PD findings according to ADA status, nor in the incidences of TEAEs or AESIs in ADA-positive vs ADA-negative patients between treatment groups at each post-baseline sample collection.

**Table 4.** Overall ADA and NAb incidence

Time point	ADA incidence*^a^* % (*n*/N*)		ADA titer Median		NAb incidence*^a^* % (*n*/N*)	
	FKS518 (*N* = 277)	The reference denosumab (*N* = 276)	FKS518 (*N* = 277)	The reference denosumab (*N* = 276)	FKS518 (*N* = 277)	The reference denosumab (*N* = 276)
52-week treatment period*^b^*	1.1 (3/274)	2.2 (6/276)	—	—	0.4 (1/274)	0.4 (1/276)

Analysis set: safety.

At least one positive post-dose result in the ADA specificity assay or the NAb assay associated with that positive ADA specificity result any time during the study period resulted in the participant being considered ADA- or NAb-positive, respectively.

Abbreviations: ADA, antidrug antibody; n, number of participants with positive status; N, number of participants in the safety analysis set; N*, number of patients with a valid ADA result; NAb, neutralizing antibody.

^*a*^A subject had an overall post-dose positive status (ADA or NAb) when having ≥1 positive result post-dose.

^*b*^Determined across all time points except Baseline (predose).

## Discussion

Over the past 2 decades, biologics have revolutionized the treatment of a range of diseases [28]; however, they have high costs and limited access in some countries [17]. Biosimilars can expand access and usage of biologics, achieving subsequent improvement in patient outcomes while leading to cost savings [19, 20]. In 2018, it was estimated that the introduction of biosimilars could reduce spending on biologics in the United States by $54 million over the period 2017 to 2026 [18].

This phase 3 randomized controlled trial demonstrated the therapeutic equivalence of the proposed denosumab biosimilar, FKS518, and the reference denosumab in efficacy and PD characteristics in postmenopausal women with osteoporosis. All sensitivity analyses, supportive estimands, secondary efficacy, and PD endpoint analyses consistently pointed to similarity between the 2 products.

Safety outcomes did not reveal any concerns and were consistent with the known safety profile of denosumab. The most common non-COVID-19 TEAEs were as expected for denosumab-treated women with postmenopausal osteoporosis, and the safety profile of FKS518 was similar to that of reference denosumab. Fractures occurred very infrequently in either treatment group during the core treatment period.

Another key assessment for the demonstration of biosimilarity is the establishment of similarity of immune response [22]. The overall incidence of ADA-positive or NAb-positive samples was low and similar between the FKS518 and the reference denosumab groups, and recorded ADA titers were low. Importantly, no meaningful clinical impact of immunogenicity was observed.

This study had several strengths. The studied treatment indication, postmenopausal osteoporosis, is generally considered the most sensitive for detecting differences between a proposed denosumab biosimilar and its reference product. The primary efficacy assessment utilized changes in LS-BMD, which correlates with fracture risk making it the most widely used measure to assess the efficacy of osteoporosis treatments. A duration of 12 months with 2 treatment cycles was considered appropriate for demonstration of efficacy equivalence, as it represents a time point when increases in BMD are measurable but have not yet plateaued. Additionally, the evaluation of BMD measurements was centralized, reducing variability in the efficacy endpoints. The study also had a strong emphasis on biomarkers, with both serum CTX and P1NP levels being assessed. Changes in the bone resorption biomarker serum CTX have a significant relationship with reduction in fracture risk, and the serum CTX assay is well characterized and has high specificity, availability and ease of use, making it a preferred PD parameter to detect potential differences between 2 denosumab products [29]. Importantly, analyses of both efficacy (change in LS-BMD) and PD (AUEC_(0-W26)_ CTX) were powered with prespecified margins to demonstrate similarity. This study therefore benefits from the complementary advantages of both measures. Study limitations included that, as is required for biosimilarity studies, the study population was homogeneous, limiting the generalizability of findings to the full population at risk of osteoporosis. In addition, no mandatory, prescheduled imaging scans were performed to identify asymptomatic fractures, so the number of reported fractures may underestimate the true number of fractures that occurred.

Denosumab has been studied extensively, and its safety and effectiveness has been demonstrated [30-32]. Changes in BMD and biomarkers of bone turnover at 52 weeks in the current study were consistent with those observed in the pivotal study of denosumab in postmenopausal women [29, 33]. This study demonstrated therapeutic equivalence of, and the comparable PD, safety, and immunogenicity profiles between, FKS518 and reference denosumab, completing the clinical evidence to propose FKS518 as a biosimilar to denosumab.

## Data Availability

The study datasets are not publicly available due to ongoing regulatory filing activities. Once the regulatory application is complete, data will be available from the corresponding author on reasonable request.

## Previous Presentation of This Work

Some Lumiade-3 study data has been presented at the American Society of Clinical Oncology (ASCO) Annual Meeting 2024; May 31–June 4, 2024; Chicago, IL in an abstract and poster: Sadek J, Valter I, de Souza A, Szeles P, Monnet J. A randomized, double-blind study to evaluate the efficacy, pharmacodynamics, safety and immunogenicity of FKS518 proposed biosimilar to denosumab with the originator in postmenopausal woman with Osteoporosis. J Clin Oncol 2024; 42(16, Suppl). https://doi.org/10.1200/JCO.2024.42.16_suppl.3155.

## Abbreviations

ADA: antidrug antibodies
AE: adverse event
AESI: treatment-emergent adverse event of special interest
ANCOVA: analysis of covariance
AUEC_(0-W26)_: area under the effect curve from baseline to week 26
BMD: bone mineral density
%CfB: percentage change from baseline
CTCAE: Common Terminology Criteria for Adverse Events
CTX: cross-linking telopeptide of type 1 collagen
DXA: dual-energy x-ray absorptiometry
IEC: Independent Ethics Committee
Ig: immunoglobulin
ITT: intention-to-treat
LS-BMD: lumbar spine bone mineral density
LSM: least squares mean
Nab: neutralizing antibodies
P1NP: procollagen type 1 N-terminal propeptide
PD: pharmacodynamic
PK: pharmacokinetic
PP: per protocol
RANK: receptor activator of nuclear factor-κB
RANKL: ligand of receptor activator of nuclear factor-κB
SAE: serious treatment-emergent adverse event
TEAE: treatment-emergent adverse event
